# Comparative morphology of the forewing base articulation in Sternorrhyncha compared with a representative of Fulgoromorpha (Insecta, Hemiptera)

**DOI:** 10.1007/s00435-015-0293-4

**Published:** 2015-12-22

**Authors:** Barbara Franielczyk, Piotr Wegierek

**Affiliations:** Department of Zoology, Faculty of Biology and Environmental Protection, University of Silesia, Bankowa 9, 40-007 Katowice, Poland

**Keywords:** Forewing base, Axillary sclerites, Aphids, Coccids, Psyllids, Whiteflies

## Abstract

The forewing articulation of single species from each of the four subgroups of Sternorrhyncha (Aleyrodomorpha, Aphidomorpha, Coccomorpha, Psyllomorpha) was examined by optical and scanning electron microscopy. The species were compared with a species of Cixiidae (Fulgoromorpha), as an outgroup of Sternorrhyncha. We present the results of a comparative analysis of the forewing articulation in these five groups, propose a standardized terminology and compare our findings with those previously reported. The wing base of all examined species is composed of the following structures: anterior and posterior notal wing process, first, second, and third axillary sclerites, tegula, and axillary cord. The number of elements included in the wing base and the surrounding area is the greatest in *Cacopsyllamali*, the most complicated species from Sternorrhyncha. Based on the shape of axillary sclerites and the number of elements forming the wing base environment, *Orthezia urticae* (Coccomorpha) and *Cixius nervosus* (Fulgoromorpha) are the most similar. Among Sternorrhyncha, the most similar axillaries are those of *Aphis fabae* and *Orthezia urticae*, which is congruent with existing classifications. In this paper we show that the four groups from Sternorrhyncha exhibit their own distinct wing base morphology.

## Introduction

The emergence of wings and ability to fly was a key to the evolutionary success of insects. Wing morphology was examined in an evolutionary context by Kukalovà-Peck (1978, 1991) and Rasnitsyn (1981), but most reports have tended to concentrate mainly on the course of veins (e.g. Comstock and Needham 1898; Hamilton 1972; Béthoux and Nel 2001, 2002; Béthoux 2007; Nel et al. 2011). The structure of the wing articulation in insects is a complex issue, which largely determines the ability to fly and its wing folding at rest (Chapman 2013). The flight issue was widely described by Wootton (1996, 2002) and Wootton and Kukalová-Peck (2000).

### General model of the wing articulation

**Fig. 1:**
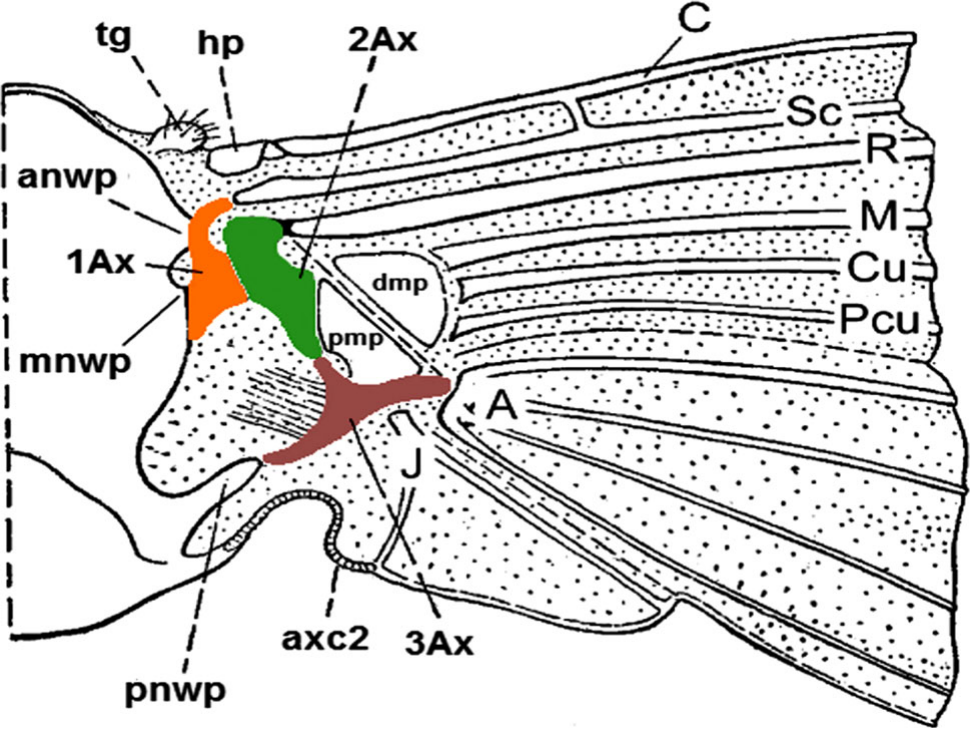
Model of the insect wing articulation (after Snodgrass 1935, modified; abbreviations in the text)

According to the diagram of the insect wing articulation (Snodgrass 1935), it usually consists of three main axillary sclerites (1Ax, 2Ax, 3Ax) [e.g. Hymenoptera and Orthoptera have a fourth axillary sclerite (4Ax) (Brodsky 1996) as also Aleyrodidae according to Weber 1935)] and the structures forming the environment of wing base. Two of these structures, the humeral plate and the tegula, constitute a connection between the wing base and the thorax. Moreover, the tegula, which is placed on each wing base (fore- and hindwing), has sensory hairs (Field and Matheson 1998). In this general model, the axillary sclerites 1Ax and 3Ax are connected to the body by lateral processes of the notum—the anterior notal wing process (anwp), the median notal wing process (mnwp) and the posterior notal wing process (pnwp) (Fig. 1). The first axillary is connected with anwp and mnwp and the third one with pnwp. The 1Ax and 2Ax are connected together. Proximal and distal median plates (pmp, dmp) can be found between the wing membrane and axillary sclerites. The dmp is connected with three veins—media (M), cubitus (Cu) and cubitus posterior (P_CU_). The whole wing pivots on the fulcrum, the dorsal tip of the pleural wing process, which is connected with 2Ax and enables the wing movements (Snodgrass 1935). The connection between the scutellum and the wing base is enabled by the axillary cord (axc2). As suggested by Hörnschemeyer (2002), all structures that form the wing articulation, including the surrounding musculature, can be used in higher-level insect phylogenetics because the wing base structure is preserved at the genus or family levels.

### The structure of the forewing articulation among insects

Many elements of the wing base are similar between the holo- and hemimetabolous insects. Within hemimetabolous insects, the wing base structure was recently examined in Hemiptera and Thysanoptera (Hörnschemeyer and Willkommen 2007), Odonata (Ninomiya and Yoshizawa 2009), and in the Dictyoptera (Yoshizawa 2011). Due to the small size of axillary sclerites, examination of the wing base has favored larger insects.

Within hemimetabolous Sternorrhyncha, there are a few studies on the course of wing veins (Patch 1909 and Klimaszewki and Wojciechowski (1993) in all Sternorrhyncha; Martin 2007 in whiteflies; Shcherbakov 2007 in aphids and coccids) and on the structure of the wings of coccids (Koteja 1996; Simon 2013).

The suborder Sternorrhyncha is divided into four infraorders: Psyllomorpha (jumping plant-lice) (Becker-Migdisova 1962), Aleyrodomorpha (whiteflies) (Chou 1963), Aphidomorpha (aphids) (Becker-Migdisova and Aizenberg 1962) and Coccomorpha (scale insects) (Heslop-Harrison 1952). Some aphid and most psyllids and whiteflies adults have two pairs of wings, while in scale insects only males have well-developed wings, and only a single pair (Gullan and Martin 2009). Most Sternorrhyncha wing base studies focused on the dorsal side of the forewing Koteja (1996) in coccids, Weber (1928, 1929) in aphids, Yoshizawa and Saigusa (2001) and Ouvrard et al. (2008) in psyllids. The forewing articulation of whiteflies was examined in *Aleyrodes proletella* Linnaeus. 1758 and both the fore- and hindwing articulation was described in *Trialeurodes vaporariorum* Westwood 1856 (Weber 1935). The forewing base structure in Fulgoromorpha, a likely sister group to Sternorrhyncha (Song et al. 2012; Song and Liang 2013) was studied by Emeljanov (1977) and Yoshizawa and Saigusa (2001).

We undertook a study (1) to re-describe and compare the forewing articulations among the representatives of Sternorrhyncha using optical and scanning electron microscopy, (2) to compare the obtained results to a representative of Fulgoromopha, (3) to compare our results with the conclusions of previous authors, and (4) to unify the terminology.

## Materials and methods

Sternorrhyncha specimens examined were of *Cacopsylla mali* (Schmidberger 1836) (Psyllomorpha), *Aphis fabae* Scopoli 1763 (Aphidomorpha), *Orthezia urticae* (Linnaeus 1758) (Coccomorpha), *Aleyrodes proletella* (Linnaeus 1758) (Aleyrodomorpha), with the sister-group represented by *Cixius nervosus* (Linnaeus 1758) (Cixiidae, Fulgoromorpha) (Table 1). These species belong to genera nominal for examined groups. The terminology of wing axillary sclerites and associated structures of the notum and pleuron follows Ouvrard et al. (2008). Additionally, Table 3 provides correspondence with the older studies of Weber (1928, 1929, 1935), Emeljanov (1977), Koteja (1986), Yoshizawa and Saigusa (2001).

**Table 1:** List of examined species

Species	Locality	Host plant	Determination data
*Aphis fabae* 20 alate females	Piekary Śląskie, Poland Bytom, Poland	*Chenopodium* sp. *Cirsium arvense*	leg. B. Franielczyk, Silesia University det. Ł. Depa, Silesia University
*Aleyrodes proletella* 20 females	Piekary Śląskie, Poland	*Chelidonium majus*	leg. B. Franielczyk, Silesia University det. J. Drohojowska, Silesia University
*Cacopsylla mali* 20 females	Goczałkowice, Poland Ustroń, Poland	*Malus* sp.	leg. B. Franielczyk, Silesia University det. J. Drohojowska, Silesia University
*Orthezia urticae* 20 males	Goczałkowice, Poland	*Urticae dioica*	leg. B. Franielczyk, Silesia University det. E. Simon, Silesia University
*Cixius nervosus* 10 females	Libusza, Poland Gładyszów, Poland	Xerothermic grasslands	leg. M. Walczak, Silesia University det. M. Walczak, Silesia University

Dry or ethanol-preserved (70 %) specimens were used. For SEM analysis, the entire insects were mounted on holders and sputter-coated with gold and examined using a scanning electron microscope Hitachi UHR FE-SEM SU 8010 (Tokyo, Japan) in the Scanning Electron Microscopy Laboratory at the Faculty of Biology and Environmental Protection, University of Silesia. The ventral part of the body, hind wings and legs were removed to facilitate observations in the light microscope. A Nikon SMZ1500 stereomicroscope was used to observe insects in glycerin. Specimens of *O. urticae* were first stained with chlorazol black following the procedure of Afifi and Kosztarab (1967). The orientation of described structures is in relation to the main axis of the body.

The abbreviations used in the text and in the figures: anwp—anterior notal wing process; 1Ax, 2Ax, 3Ax, 4Ax—axillary sclerites 1, 2, 3, 4; axc2—axillary cord; br—basiradiale; brb—basiradial bridge; bsc—basisubcostale; dmp—distal median plate; hp—humeral plate; m—mesonotum; mnwp—median notal wing process nt1—pronotum; pmp—proximal median plate; pnwp—posterior notal wing process; ppt—parapterum; prb—prealar bridge; psc2—praescutum; pwp—posterior wing process; sc2—mesoscutum; scl2—mesoscutellum; tg—tegula.

## Results

The structure of the wing base in the examined species is described below. The differences between the studied species are summarized in Table 2. A standardized terminology is given in Table 3.

**Table 2:** Elements of the wing base and their presence in the examined species from Sternorrhyncha and in *C. nervosus*

Structure	*Cacopsylla mali*	*Aphisfabae*	*Orthezia urticae*	*Aleyrodes proletella*	*Cixius nervosus*
anwp	+	+	+	+	+
mnwp					+
pnwp	+	+	+	+	+
1Ax	+	+	+	+	+
2Ax	+	+	+	+	+
3Ax	+	+	+	+	+
tg	+	+	+	+	+
hp	+	+	+	+	
bsc	+				
br	+				
dmp	+	+		+	
pmp	+				
prb	+				
brb	+			+	
ppt	+		+		
axc2	+	+	+	+	+

**Table 3:** Corresponding terminologies between elements of the wing base of representatives of Sternorrhyncha and Fulgoromorpha by previous authors with standardized one (in bold)

Weber (1928)		tg	Ax_1_	Ax_2_	Ax_3_							N_1_	Psc_2_	Sc_2_	Scl_2_	THa		THb	plFGK
Weber (1929)	Po_1_	Po_2_	Ax_1_	Ax_2_	Ax_3_			Par1			Par2	N_1_	Psc_2_	Sc_2_	Scl_2_	THa			plFGK
Weber (1935)	1Po_1_	2Po_2_	1Ax_1_	2Ax_2_	3Ax_3_	4Ax_2_		Bas			Sub	N_1_	Psc_2_	Sct_2_	Scl_2_	THa			hlFGK_2_
Emeljanov (1977)																			
Koteja (1986)																			
Yoshizawa and Saigusa (2001)		Tg	1Ax	2Ax	3Ax		HP		PMP	DMP1 DMP2						ANWP	MNWP	PNWP	
Ouvrard et al. (2008)	ppt	tg	1Ax	2Ax	3Ax		hp	bas	pmp	dmp		nt1	psc2	sc2	scl2	anwp		PNWP pnwp	
**Interpretation**	**ppt**	**tg**	**1Ax**	**2Ax**	**3Ax**	4Ax	**hp**	bas	**pmp**	**dmp**	sub	**ntl**	**psc2**	**sc2**	**scl2**	**anwp**	mnwp	**pnwp**	**pwp**

### *Cacopsylla mali* (Figs. 2a, 3a, 5a, 6a)

The pronotum (nt1) does not reach the wing base.

**Fig. 2:**
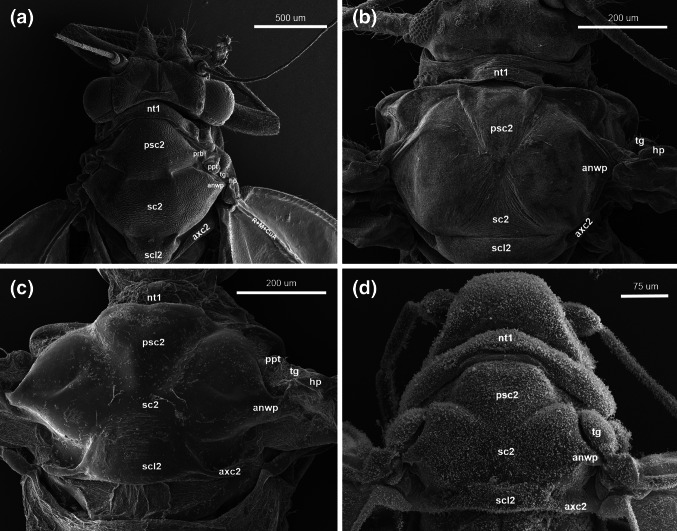
Scanning electron microscopy showing the thorax of **a** *Cacopsylla mali* **(**Schmidberger 1836), **b** *Aphis fabae (*Scopoli 1763), **c** *Orthezia urticae* (Linnaeus 1758), **d** *Aleyrodes proletella* (Linnaeus 1758), dorsal view

**Fig. 3:**
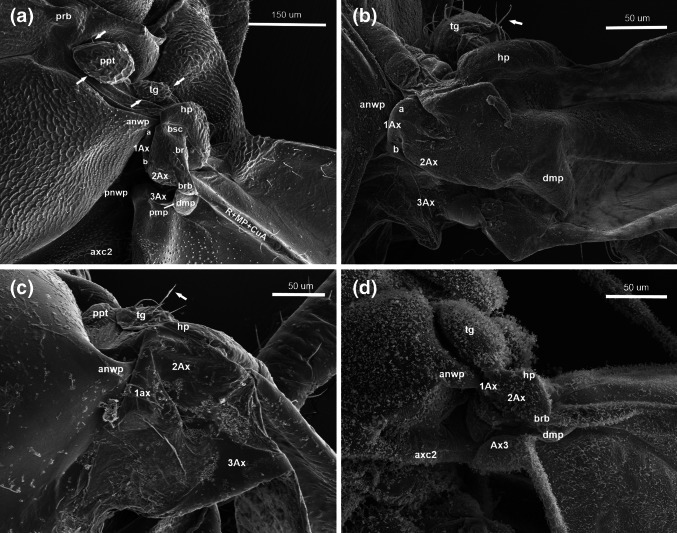
Scanning electron microscopy showing the forewing articulation of **a** *Cacopsylla mali* **(**Schmidberger 1836), **b** *Aphis fabae* (Scopoli 1763), **c** *Orthezia urticae* (Linnaeus 1758), **d** *Aleyrodes proletella* (Linnaeus 1758), dorsal view; *white arrows* indicate hairs

The praescutum (psc2), mesoscutum (sct2) and mesoscutellum (scl2) are visible. The praescutum (psc2) laterally forms a small, globular extension, a prealar bridge (prb).

The wing base is articulated with the mesonotum by two processes: the upper one, anterior notal wing process (anwp) and the lower one, posterior notal wing process (pnwp) (Fig. 5a).

Two bulge-like structures are visible under prb: a bigger parapterum (ppt) and a smaller tegula (tg). Both are covered with a few small hairs (Figs. 3a white arrows, 5a).

The ligament-like axillary cord (axc2) runs laterally, parallel to the scutum (Fig. 2a).

**Fig. 4:**
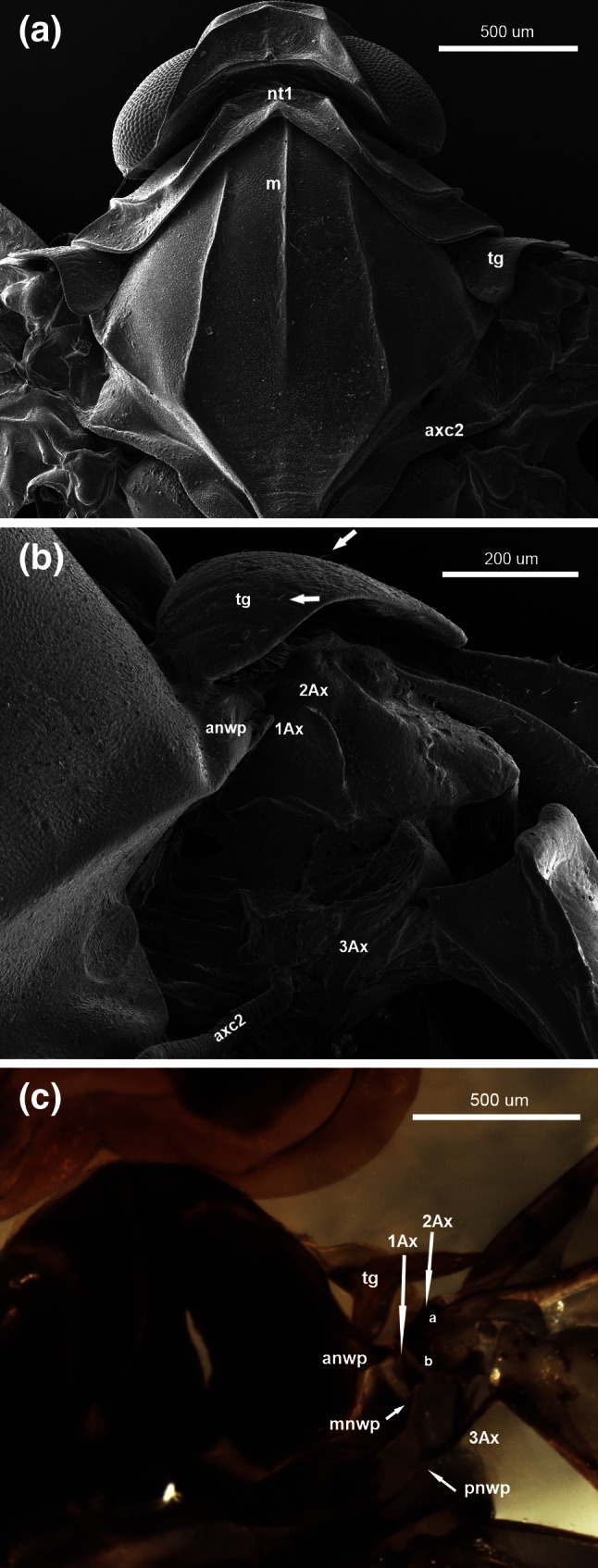
Scanning electron microscopy showing **a** the thorax of *Cixius nervosus* (Linnaeus 1758), **b** the forewing articulation of *Cixius nervosus* (Linnaeus 1758); optical microscopy showing **c** the forewing articulation of *Cixius nervosus* (Linnaeus 1758), dorsal view

**Fig. 5:**
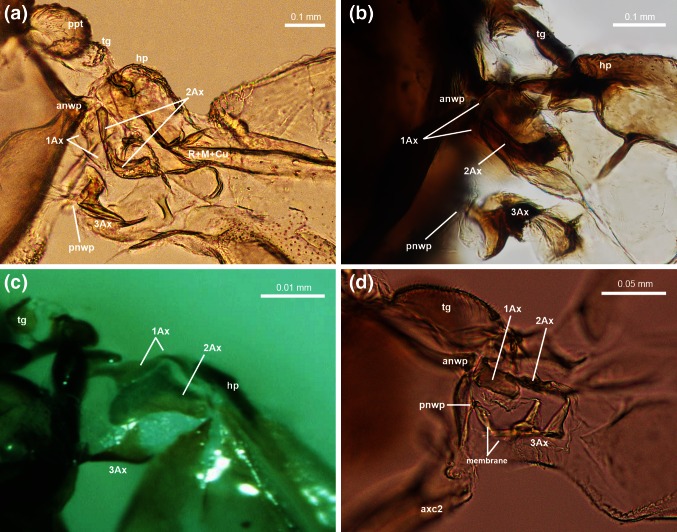
Optical microscopy showing the forewing articulation of **a** *Cacopsylla mali* **(**Schmidberger 1836), **b** *Aphis fabae* (Scopoli 1763), **c** *Orthezia urticae* (Linnaeus 1758), **d** *Aleyrodes proletella* (Linnaeus 1758), dorsal view

The forewing articulation consists of three axillaries (Fig. 5a).

The first axillary sclerite articulates with the anterior notal wing process by an indentation on the top of its anterior arm (a), runs along the lateral edge of scutum and by its posterior arm (b), adjoins the second axillary sclerite (Fig. 3a).

The second sclerite also articulates with the anwp. The posterior part of this sclerite has the shape of acetabulum and is directed to the main wing vein (R + MP + CuA) (Fig. 5a). 1Ax is almost entirely hidden by the second sclerite.

**Fig. 6:**
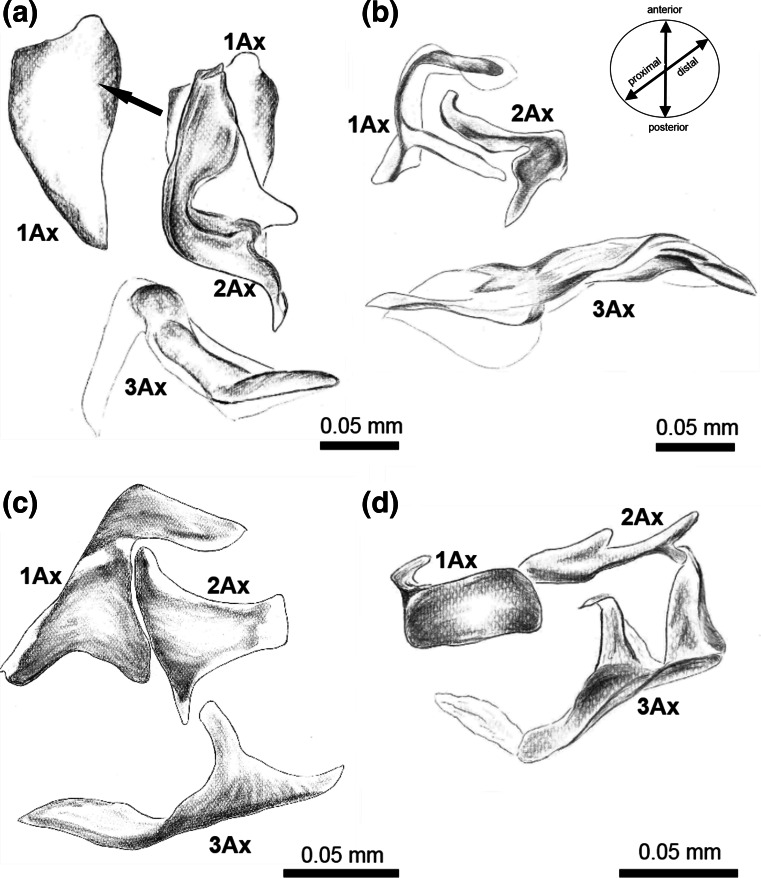
Schematic drawing showing shapes and relations between axillary sclerites of the forewing articulation of **a** *Cacopsylla mali* **(**Schmidberger 1836) (an additional drawing of a separated sclerite 1Ax), **b** *Aphis fabae* (Scopoli 1763), **c** *Orthezia urticae* (Linnaeus 1758), **d** *Aleyrodes proletella* (Linnaeus 1758), dorsal view

The first sclerite is triangular, with more sclerotized margins, while the second one is L-shaped (Fig. 6a). The central part of each sclerite is filled with membrane, which is also more or less sclerotized than the margins. 3Ax is curved in shape and strongly sclerotized halfway, the remaining area is membranous (Fig. 6a).

The third axillary sclerite adjoins pnwp (Fig. 5a).

Another extension, called humeral plate (hp), is visible below the tegula (Fig. 3a). This structure is fused with the basisubcostale (bsc), the proximal end of the costal + subcostal (C + Sc) vein, which is situated below.

The bsc is connected with the basiradiale below (br), situated on the basal part of the radial vein.

The connection between 2Ax and R + MP + CuA (=traditional R + M + Cu) vein is provided by a basiradial bridge (brb), which covers the basiradiale.

A small, rounded distal median plate (dmp) is located below brb; it has visible proximal median plate (pmp) on its proximal edge (Fig. 3a).

### *Aphis fabae* (Figs. 2b, 3b, 5b, 6b)

Pronotum (nt1) does not reach the wing base (Fig. 2b).

Prescutum, mesoscutum and mesoscutellum are visible.

The anterior notal wing process (anwp) and the posterior one (pnwp) are present (Fig. 5b).

The globular tegula and flat humeral plate are visible and the first one in covered with short hairs (Fig. 3b, white arrows).

Parapterum is not present.

The distal median plate, almost triangular in shape, is clearly visible (Fig. 3b).

A ligament-like axillary cord (axc2) runs laterally, parallel to the scutum (Fig. 2b).

1Ax has two long arms, the anterior one (a) runs along the notum, connects with anwp and is directed to sclerotized subcostal vein, which it finally joins and the posterior arm (b) connects with 2Ax (Fig. 3b). This sclerite is longitudinal, adjacent to 1Ax.

The last sclerite, 3Ax, is slightly twisted and directed to the body with a forked end (Figs. 5b, 6b).

### *Orthezia urticae* (Figs. 2c, 3c, 5c, 6c)

Pronotum (nt1) does not reach the wing base (Fig. 2c).

Prescutum, mesoscutum and mesoscutellum are visible.

The anterior notal wing process (anwp) is clearly visible and joins 1Ax (Fig. 3c).

The posterior notal wing process (pnwp) is present but visible only when the wings are raised.

The anterior structure called parapterum (ppt) is almost entirely hidden by tegula.

The tegula resembles a roofing tile and slightly covers forewing articulation. It is covered with a few hairs (Fig. 3c, white arrows).

The humeral plate is present distally (Fig. 3c).

The axillary cord runs parallel to the scutum (Fig. 2c).

Axillaries are more or less triangular in shape but sometimes only in outline. 1Ax has the shape of an equilateral triangle and its anterior tip is curved around the anterior end of 2Ax.

The second sclerite is less obviously triangular with four projections.

The first one is surrounded by 1Ax, the second is directed toward subcostal vein, the third is connected to the wing membrane and the last one is directed to 3Ax.

The third axillary sclerite, which is more isosceles triangle-like in shape, is twisted about 180° in the proximal part when the wings are directed downwards (Figs. 5c, 6c). It is proximally connected with pnwp and distally with the anal vein.

### *Aleyrodes proletella* (Figs. 2d, 3d, 5d, 6d)

Pronotum (nt1) almost reaches the wing base (Fig. 2d).

Prescutum, mesoscutum and mesoscutellum are visible (Fig. 2d).

1Ax articulates with anwp (Fig. 3d).

The posterior notal wing process is recognizable as a posterior articulation of 3Ax (Fig. 5d).

Two external extensions, the tegula and the humeral plate, are visible. Tg is oval and well formed. Hp is a small plate below tg (Fig. 3d).

Parapterum is not present.

The basiradial bridge is located near the main wing vein (Fig. 3d).

There is a small, triangular extension below the basiradial bridge, probably the distal median plate (dmp).

The first axillary sclerite is the biggest, rather trapezoid in shape, with a small tip on the upper edge directed to the wing membrane. The body of 1Ax is connected to 2Ax, the elongated sclerite directed toward the wing membrane.

The last one, 3Ax, is composed of an elongated body terminating with a triangle and having one triangular outgrowth in the middle. The ending is joined to 2Ax (Figs. 5d, 6d). There is a membranous element between the body and 3Ax, which links these two elements and is adjacent to the posterior notal wing process (Fig. 5d).

### *Cixius nervosus* (Figs. 4a–c, 7b)

Collar-shaped pronotum (nt1) is well developed and reaches down almost the forewing articulation (Fig. 4a).

**Fig. 7:**
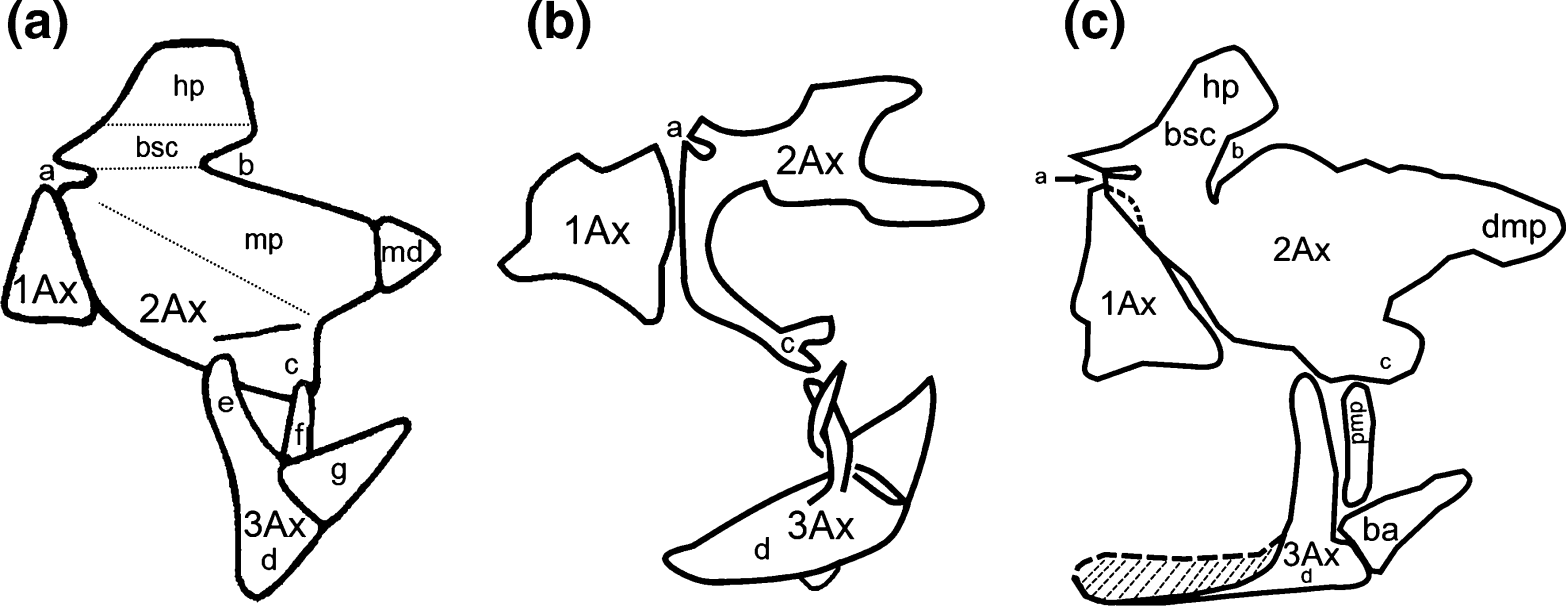
Schematic drawings of axillary sclerites of *Cixius nervosus* (Linnaeus 1758) **a** modified, after Emeljanov 1977, **b** present interpretation, **c** *Oliarus angusticeps* (Horváth 1892) modified, after Yoshizawa and Saigusa (2001)

Anwp joins 1Ax and pnwp is directed toward 3Ax (Fig. 4c).

The tegula is enlarged with a broad extension surrounding the entire outer margin of the wing base. Its surface is rather smooth with a few small hairs (Fig. 4b, white arrows). Parapterum is not present.

Axillary cord (axc2) runs parallel to the posterior edge of mesonotum (m).

1Ax resembles a small trapezoid, with four clearly defined edges. The proximal edge is connected with anwp. Two distally located edges form a wall parallel to 2Ax. The fourth one is directed to the process of notum, mnwp (Fig. 4c).

2Ax has two arms; the longer but less sclerotized one (a) extends toward the wing membrane and the shorter but more sclerotized one (b), with a forked end, is directed to the common stem of veins ScP + R + MP + CuA (Fig. 4c). Between two arms of 2Ax a small indentation (a) is visible (Fig. 7b).

The third axillary sclerite is build of a longitudinal body with two outgrowths (Fig. 7b). The body of this sclerite is directed toward the claval edge of the wing distally and connects with the posterior notal wing process (pnwp) proximally (Fig. 4c).

## Discussion

### Axillary sclerites

The five examined species possess three axillary sclerites (1Ax, 2Ax, 3Ax). Differences concern mostly the shape of the sclerites. According to Weber (1935), in *A. proletella* 3Ax has two outgrowths, but the first, proximal one, is additionally forked. Our observations did not confirm that interpretation, and we also found a larger 2Ax than did Weber (1935). Our results for *O. urticae* were similar to those of Koteja (1986) with respect to all three axillary sclerites, with one exception: the position of the axillary sclerites was the same but only when the wing is directed downwards, as shown in Fig. 5c. In turn, in *A. fabae,* Weber (1928) described 1Ax as connected by its distal, upward curved end with an also curved ending of the subcostal vein and by its thickened distal part with 2Ax. The second axillary sclerite was diamond-shaped and constituted a connection between 1Ax and 3Ax. Our interpretation is similar: 1Ax has two arms, as was described earlier. The upper one is connected to the subcostal vein and the lower one to 2Ax. The second axillary sclerite is more elongated in shape than diamond-like but lies very close to 1Ax.

Emeljanov (1977), who described the overall pattern for Fulgoromorpha in general, presented 1Ax as an equilateral triangle (Fig. 7a) which in our opinion is more trapezoid in shape (Fig. 7b). But it is the second sclerite that proves most problematic. Emeljanov (1977) showed 2Ax as a sclerite composed of several fused elements (Fig. 7a). According to that author, the anterior part is formed by a humeral plate (hp), which is connected below to the basisubcostale (bsc). Two indentations are marked at this location: proximal anterior (a) and distal posterior (b). The latter lies at the height of the Sc + R + M vein. Below, a wider part of the sclerite is composed of the element called 2Ax and of the median plate (mp), connected with the small triangular distal plate (md). Distally, 2Ax ends with a distal process (c), the connection with 3Ax. The second axillary sclerite is connected with 1Ax by its straight anterior external edge. Yoshizawa and Saigusa (2001) described the second sclerite in Fulgoromopha as irregular and comprising a few elements: the upper, proximal part of 2Ax is fused with the basisubcostale and humeral plate without clear boundaries and the distal part passes smoothly into the distal median plate (dmp = md) and at the bottom almost links 3Ax and pmp (c). Authors did not mention about it, but we can point out the two indentations (a and b), which Emeljanov (1977) showed earlier (Fig. 7c). Our description of 2Ax is somewhat different (Fig. 7b), but agrees with Emeljanov’s (1977) in the following: there is a proximal anterior indentation (a); also the posterior edge and a distal process (c) are clearly visible. Other elements cannot be distinguished because the rest of sclerite is membranous; only both arms of 2Ax are strongly sclerotized and easy to find in the wing articulation under the light microscope. Yoshizawa and Saigusa (2001) indicated that the third sclerite has a long body (d) extending from pnwp, with one branch facing 2Ax. Another two elements connected to the sclerite body are basanale and pmp; however, they said that pmp is not always present. According to Emeljanov (1977), 3Ax has a short body (d) with two outgrowths (e, f), connected with 2Ax and one separated distal outgrowth (g). The latter is directed toward the anal vein and connects to the jugal part of the wing membrane. Our results indicate that the 3Ax has a sharply pointed longitudinal body (d) and two appendages growing out of the sclerite body and rolled up in opposite directions. Both are directed toward 2Ax (Fig. 7b). It is very difficult to see any homologies between outgrowths e and f form Emeljanov (1977) work and two appendages from present study. In our interpretation those two elements are twisted around each other and the whole 3Ax sclerite seems to be turned so only indication of differences is possible here.

### Connections between axillaries

The general pattern of axillary sclerites is the same for all the examined Sternorrhyncha. The first sclerite joins anwp and 2Ax; the second one is linked with 1Ax and distally is directed to the main, central vein; the third one is always connected with pnwp and with the wing membrane, near the anal vein. Nevertheless, the connections between axillary sclerites seem important. According to Yoshizawa and Saigusa (2001), in Cixiidae 1Ax is connected proximally to both anwp and mnwp and distally to 2Ax. They only found a small gap between those two sclerites. According to Emeljanov (1977), all sclerites in the representative of Cixiidae are very closely connected as if they were fused. Our research confirmed the relation between 1Ax/notum and 1Ax/2Ax as presented by Yoshizawa and Saigusa (2001) and also showed that between 1Ax and 2Ax there is a clearly visible space where the sclerites are connected to each other by a thin membrane (Figs. 4c, 7c). Because of the connection between 1Ax and anwp and mnwp, the arrangement of axillary sclerites in *C. nervosus* is most similar to the general pattern of wing articulation presented by Snodgrass (1935). The connection between 1Ax and 2Ax, which are very close to each other in psyllids, was described by Ouvrard et al. (2008). Our observations confirm that report. Ouvrard et al. (2008) also noted the lack of connection between 2Ax and 3Ax. Likewise, we are convinced that there is no sclerotized connection but only a membranous one. This is in contrast to Weber (1929) and Yoshizawa and Saigusa (2001), who did not indicate a separation between them. Besides, according to our study, both the first and second axillary sclerites have their origin in anwp, which is unusual in the examined Sternorrhyncha. Axillary 1Ax is hardly visible. The relations between three sclerites in *O. urticae* described by Koteja (1986) are similar to our results, i.e. 1Ax surrounds 2Ax and the latter is connected with 3Ax.

The wing base in *A. proletella* is the hardest to interpret. As noted by Weber (1935), this part of the Aleyrodidae body is small and difficult to examine. Regardless, our results are not compatible with Weber’s (1935): we could not confirm the presence of 4Ax. After preparation, there seemed to be only a slightly sclerotized membranous part of 3Ax (Fig. 6d). In *A. fabae* there is an articulation between 2Ax and 3Ax and the latter proximally extends between the notum process and distally between 2Ax and the anal part of wing. However, the connection between 2Ax and 3Ax is not as pronounced as suggested by Weber (1928). These two sclerites are connected only by a thin membrane.

### Other elements of the forewing base

The tegula is more or less variable but present in all the examined species. It has a globular form in *C. mali*, *A. fabae* and *A. proletella* or is a sclerite covering the wing base from the top as in *O. urticae* and *C. nervosus.* This sclerite is always covered by a few short hairs, even in *A. proletella* under the wax covering. In his work on morphology of Ortheziidae, Koteja (1986) pointed out that tegula is composed of two parts. According to our observations under the optical microscope, these are two different elements: the lower one is the tegula, covered by hairs and having clearly visible edges, and the upper one should be considered as parapterum (Fig. 3c). In turn, Weber (1935) reported 1Po_2_, in Aleyrodidae, which was a flat, frontal cushion on the front part of the lateral edge of the scutum. We could not identify such a structure in our studies. The second one, 2Po_2_, the rear cushion is, according to Weber (1935), identical with the tegula and we can consent to it. It is a domed part of the scutum over the wing base, so it can be interpreted as the tegula. A similar condition occurred in *C. mali* (Weber 1929), where two terms Po_1_ and Po_2_ were seen. In our study these are referred to parapterum and tegula, respectively. Muscle attachments, the subalare and basalare structures (Hörnschemeyer and Willkommen 2007), are visible only in the lateral view. Therefore, they could not have been recognized in our study in the dorsal aspect in *A. proletella*, *O.* *urticae* and *C. mali,* as indicated by Weber (1935), Koteja (1986) and Ouvrard et al. (2008), respectively. The humeral plate, located near the costal vein, is visible as a thickening or convexity and occurs in all the examined Sternorrhyncha species. Previously, it was reported only by Ouvrard et al. (2008) in psyllids. While describing *O. urticae*, Koteja (1986) wrote about “the costal complex”, which, in our opinion, should rather be called a humeral plate. According to Emeljanov (1977), hp is fused with other elements that form 2Ax; also Yoshizawa and Saigusa (2001) indicated hp in Cixiidae (Fig. 7c). However, the occurrence of these elements could not be confirmed in our study. The distal and proximal median plates were for the first time described only for psyllids within Sternorrhyncha (Ouvrard et al. 2008); we found the former in *A. fabae* and *A. proletella* and the latter only in psyllids. The prealar bridge is only visible in *C.* *mali*; it is a long and narrow process extending downwards and slightly backwards to the mesoepisternum, as described Ouvrard et al. (2008). The connection of basiradiale with the distal median plate is wrapped around the central vein and referred to as a basiradial bridge; it is recognized in *O. urticae* and *A. proletella*. The parapterum, as mentioned above, is easy to find in *O. urticae* and *C. mali*—it is an extension situated above the tegula. The axillary cord (axc2) is recognizable in all the examined species. Previously, it was described only in Psyllomorpha by Ouvrard et al. (2008). The same authors pointed out that it was still doubtful whether pnwp belonged to the scutum or the scutellum. After Resh and Cardé (2003), the anterior notal wing process is defined as an anterior lobe of the lateral margin of the alinotum supporting 1Ax, and the posterior notal wing process as a posterior lobe supporting 3Ax. It seemed that pnwp constituted a part of the scutum. In our results, the posterior notal wing process is a part of the axillary cord and, accordingly, we have interpreted it as a part of the scutellum.

### Relationships inferred from the wing base structure

There are four independent directions specific to each group. One noticeable tendency is that the first axillary sclerite, in all species, has a curved top outgrowth, which in Fulgoromorpha is not developed. The second and third axillary sclerites in *A. fabae, A. proletella* and *C. mali* differ in shapes among each other and are remarkably divergent from those in *C. nervosus*. The similarity in shape of each sclerite is most evident between the primitive coccid, *O. urticae* and *C. nervosu*s. A small number of structures forming the environment of the wing base are alike as well. The species differ in: the shape of 1Ax (triangular with a curved tip in *O. urticae* and more trapezoid in *C. nervosus*), the shape and size of 2Ax (triangle in *O. urticae* and crooked in *C. nervosus*) and the structure of 3Ax (an elongated body of sclerite with one outgrowth in *O. urticae* and an elongated body with two curved outgrowths in *C.* *nervosus*). Coccids are known as the weakest flyers (Gullan and Martin 2009) so a highly advanced wing articulation is not necessary for them. They fold wings flat over the abdomen. The other species we examined fold their wings roof-like (synonym tent-like), but in *A.* *fabae* and *C. mali* we can see little apical overlap of the forewings. In *A. proletella* and *C. nervosus*, there is no apical overlap of the forewing so it looks like folding wings flat when in their resting position. It possibly explaining the different shape of the axillary sclerites in these species in comparison with those of *O. urticae* (Dolling 1991). Additionally, in *O. urticae*, *A. proletella* and *C. nervosus*, the second and third axillary sclerites are very close to each other and almost connected, which is also relevant to their wing position at rest.

We presented four different types of wing base morphology that can be compared to Sternorrhyncha molecular analyses phylogenies. The most common morphological view on Sternorryncha phylogeny recognizes a monophyletic group consisting of aphids + coccids and psyllids + aleyrodids as sister groups (Hennig 1981; Carver et al. 1991). Analyzing the layout of axillary sclerites (Fig. 6), we can risk the statement that the most similar sclerites are between aphids and coccids (Fig. 6b, c). It is likely that sclerites of *O.urticae* became more membranous with only edges strongly sclerotized and changed into elements occurring in *A. fabae*. Research on additional species of each group is required to adequately validate this hypothesis. On the other hand, analysis of Table 4 confirms all mentioned relations. Characters and their states collected in this table summarize the information about of axillary sclerites and connections between them. After detailed analysis, we can say that on the base of some features there is similarity between aphid + coccids and between psyllids + aleyrodids.

**Table 4:** Axillary characters and their states in Sternorrhyncha infraorders

	Psyllomorpha	Aphidomorpha	Coccomorpha	Aleyrodomorpha
Anterior tip of 1Ax curved around anterior end of 2Ax	Not present	Present	Present	Not present
2Ax does not overlap 1Ax	False	True	True	True
3Ax with at least one outgrowth	Not present	Not present	Present (with one outgrowth)	Present (with two outgrowths)
Tegula	Large, globular	Large, globular	Flat, small	Large, globular
Humeral plate	Tubercle-like	Flat	Flat	Tubercle-like
Parapterum	Tubercle-like	Not present	Flat	Not present
Connection between 1Ax/2Ax	Present	Present	Present	Present
Connection between 2Ax/3Ax	Not present	Not present	Present	Present

Based on results obtained by Ouvrard et al. (2008), we can conclude that the general plan of the wing base is specific for each group within Sternorrhyncha, but based on morphological features, we can try to infer the phylogenetic relationships.
